# Using Fuzzy Multiple Criteria Decision-Making Approach for Assessing the Risk of Railway Reconstruction Project in Taiwan

**DOI:** 10.1155/2014/239793

**Published:** 2014-03-18

**Authors:** Shih-Tong Lu, Shih-Heng Yu, Dong-Shang Chang

**Affiliations:** ^1^Graduate Institute of Project Management, Kainan University, No. 1 Kainan Road, Luchu, Taoyuan 33857, Taiwan; ^2^Department of Business Administration, National Central University, No. 300 Jhongda Road, Jhongli, Taoyuan 32001, Taiwan

## Abstract

This study investigates the risk factors in railway reconstruction project through complete literature reviews on construction project risks and scrutinizing experiences and challenges of railway reconstructions in Taiwan. Based on the identified risk factors, an assessing framework based on the fuzzy multicriteria decision-making (fuzzy MCDM) approach to help construction agencies build awareness of the critical risk factors on the execution of railway reconstruction project, measure the impact and occurrence likelihood for these risk factors. Subjectivity, uncertainty and vagueness within the assessment process are dealt with using linguistic variables parameterized by trapezoid fuzzy numbers. By multiplying the degree of impact and the occurrence likelihood of risk factors, estimated severity values of each identified risk factor are determined. Based on the assessment results, the construction agencies were informed of what risks should be noticed and what they should do to avoid the risks. That is, it enables construction agencies of railway reconstruction to plan the appropriate risk responses/strategies to increase the opportunity of project success and effectiveness.

## 1. Introduction

In the past few decades population densities rapidly increased, and the quality of living much improved in urban area because of blooming economy, which results in great demand on public transportation infrastructure services in urban and metropolitan areas. The developments of public transportation infrastructures in urban and metropolitan areas tended to be undergrounded or elevated because of the limit of land use. The underground and elevated transportation system could benefit from reducing traffic effects during construction period and significantly improve the traffic after completion. Moreover, the environmental pollutions are reduced, the city appearances and the quality of living are improved, and the land utilization and the level of economic activities were also enhanced. Therefore, Taiwan's government plans to reconstruct the railway systems in underground or elevated structure for several urban areas in Taiwan in recent years, and the expected total budget amount of these developments reaches about NT$140 billion [1].

Currently, the railway reconstruction projects of Taiwan have been kickoff in several highly populated urban areas and encountered tons of expected and unexpected challenges, because railway reconstruction projects are performed under a series of communication, coordination, and cooperation to integrate many works and arrange complicated interfaces in a limited working area. Therefore, carelessness during construction works or inadequate plan could lead to occurrences of accidents and cause great damage of lives, assets, environment, and the society [2]. Railway reconstruction work usually has the following challenges: (1) the limitations of available working areas, especially to the management of machineries, materials, personnel, and dynamic access; (2) remaining the normal operations of original railway system and maintaining safety at work areas; (3) managing fluent traffic flow nearby construction sites; (4) remaining the functions of the pipelines in or nearby construction sites through the temporary works of hoist, reroute, or transfer; (5) protecting nearby residential buildings and facilitating as well as managing safety of residents and pedestrians nearby job sites; (6) using customized equipment to perform works on the job areas with restricted spaces such as height limitations under existed bridges; (7) corresponding to current regulations of environment protection, noise, vibration, and air and water pollutions [1]. These challenges represent a variety of risk factors in the railway reconstruction projects.

In addition Datta and Mukherjee [3] mentioned that an underground railway project is highly complex and has many potential risks. Those potential risks frequently lead to great losses to the clients (i.e., the railway reconstruction agencies) and the general contractors. Therefore, the adoption of risk management strategy should be indispensable for the clients and general contractors. In other words, the risk associated with the railway reconstruction project can be reduced or even eliminated by the systematic process involving risk identification, risk assessment, analysis, and selection of appropriate risk management solutions. When risks and risk factors were identified, they can be effectively managed by reducing their occurrence probability or decreasing the associated effects of risk events. Risk assessment plays a core role to link identified risk factors and associated risk responses and is a very complicated and difficult work in the risk management process. Therefore, to improve the project success for railway reconstruction projects a scientific, easy operated risk assessment model should be developed to serve a basis to assist decision-making for clients and general contractors.

Bellman and Zadeh [4] developed a novel method to improve decision-making in a fuzzy environment which provides a guideline to apply the fuzzy set theory [5] to investigating uncertain problems. Many past researches have implemented this approach to their multiple criteria studies, such as Kangari and Riggs [6], Paek et al. [7], Tah and Carr [8], Lu and Tzeng [9], Rebiasz [10], and Sadiq et al. [11]. However, risk assessment of a railway reconstruction project should consider several potential aspects, for example, economy trends driving the fluctuations of material and labor prices, finance problem influencing the project schedule and cost, operation gaps with project plan leading to project chaos and political interrupts leading to scope change, and so forth. Therefore, risk assessment for a railway reconstruction project is a multiple criteria problem. This study adopts Fuzzy MCDM which combines multiple criteria decision-making (MCDM) techniques and fuzzy set theory to help decision makers to assess the occurrence likelihood of risk factors and their impacts on project success for railway reconstruction projects. First, the study identified the potential risk factors in railway reconstruction project through literature reviews and further classified the identified factors. Secondly, a questionnaire with 7 scales of linguistic variables was developed to evaluate the degrees of impact and occurrence likelihood of identified risk factors by investigating experts on railway reconstruction field. Finally, the risk assessment model was suggested.

## 2. Risk Assessment Model

Tah and Carr [12] describe the process of risk management including risk identification, risk assessment, risk control and monitoring, and feedback. The purpose of this study focuses on establishment of risk assessment model. In advance of model assessment, a hierarchical structure of risk factors should be investigated.

### 2.1. Constructing Hierarchical Structure of Risk Factors

Many literatures and technical reports associated with the identification and management of risks in construction projects were reviewed in this study. Chapman [13] defined the sources of risk which comprise environment, industry, clients, and project and identified 58 related risk factors. Tah and Carr [14] constructed a hierarchical structure of construction risk factors, classified into two categories: external risk and internal risk. Shen et al. [15] divided construction risk into 6 dimensions: financial, legal, management, market, policy, and political and technical. Faber and Stewart [16] pointed out the reasons of accidents in construction project including unsafely man-induced factors, unsafely physical factors, and unpredicted or force majeure. Baloi and Price [17] explored the influential factors of project cost performance as the risk factors of project, including estimator related, design related, level of competition related, fraudulent practices related, construction related, economic related, and political related. Ghosh and Jintanapakanont [18] classified the risk factors into 9 major types, including financial and economic risks, contractual and legal risks, subcontractors-related risks, operational risks, safety and social risks, design risks, force majeure risks, physical risks, and delay risks. Öztaş and Ökmen [19] identified 14 risk factors, which contained risks associated with changes in quality and scope of work, design changes, delays in design, third party delays and defaults, bureaucratic problems, exceptionally inclement weathers, owner delays, difficulties/delays in the availability of materials, equipment and labor, poor work quality and the needs for correction, unforeseen ground conditions, inflation, exchange rate fluctuations, accidents, and inadequate specifications. Bing et al. [20] explored 13 factors for construction project risk, including political and government policy, macroeconomic, legal, social, natural, project selection, project finance, residual risk, design, construction, operation, relationship, and the third party levels. Öztaş and Ökmen [21] classified construction project risks into risks associated with defective designs, design changes, subcontractors' defaults, fluctuations in labor productivity, delays in resolving disputes, promoter delays, difficulties/delays in the availability of materials, equipment and labor, poor work quality and the needs for correction, changes in quantity, and the scope of work. Zou et al. [22] classified risks into cost-related risks, time-related risks, quality-related risks, environment-related risks, and safety-related risks. Lam et al. [23] suggested 5 risk dimensions for a project, that is, capability, contractual and legal, economic, physical and political, and societal. The risks associated with the capability dimension include designs by contractors, errors of subcontractors, operational quality, site safety, and approvals from authorities. The contractual and legal dimension includes conflicting documentation and third party delays. The economic risk dimension includes inflation and availability of labor and equipment. The physical dimension includes ground situations, access to sites, variations in number, and variations in weathers. The political and societal risk dimension includes alterations of regulations, disruption of public order, industrial disputes, and strikes. Zou et al. [24] classified construction project risk factors into clients-, designers-, contractors-, subcontractors/suppliers-, and government agencies-related risks and risks associated with external issues. Dikmen et al. [25] graphed project risks including construction risks, design risks, payment risks, client risks, subcontractor risks, and risks associated with contract clauses, wherein the construction risks include technical risks, managerial risks, resource risks, and productivity risks. Zayed et al. [26] identified four macrolevel risk areas: finance, political, culture, and market and seven microlevel risk areas: technology, contract and legal issues, resources, design, quality, construction, and others (weather, physical damage). Luu et al. [27] identified 16 factors through a questionnaire survey of 166 professionals to quantify the probability of construction project delays in a developing country. The sixteen factors were grouped into five categories that are materials, consultants, contractors, owners, and construction environment. Mojtahedi et al. [28] extend the concept of safety to risk identification and construct potential risk in gas refinery plant construction case study, including international relations, inflation, subcontractor interferences, and changes in rates of exchange. 30 risk factors. Zavadskas et al. [29] divided project risk into three groups: external risk, project risk, and internal risk. External risk includes political risk, economic risk, social risk, and weather risk. Project risk includes time risk, cost risk, work quality, construction risk, and technological risk. Internal risk includes resource risk, project member risk, construction site risk, documents and information risk, stakeholders' risks, designers risk, contractor risk, subcontractor risk, and team risk. Nieto-Morote and Ruz-Vila [30] constructed a hierarchical structure of risks for rehabilitation project of a building including project management risks: lack of adequate process, lack of resources, inexperienced team members, and lack of motivation attitudes, engineering risks: design errors and design changes, execution risks: mistakes of construction, low productivity, lack of previous experiences, and accidents, and supplier risks: technical problems, delays in supply, and lack of quality.

**Figure 1:**
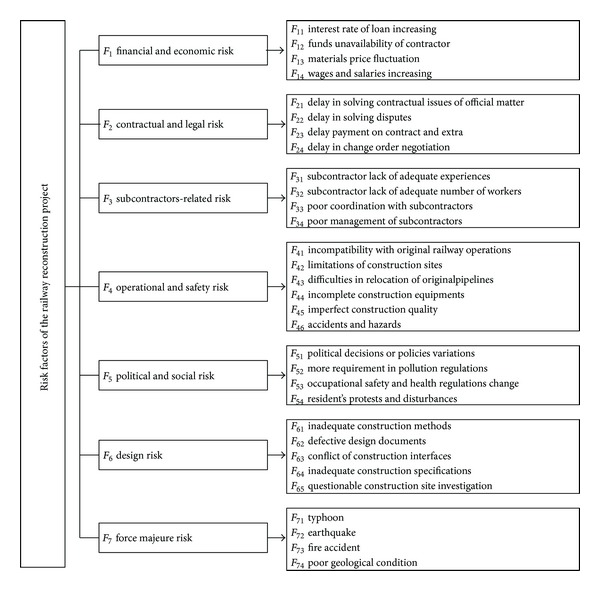
Hierarchical structure of risk factors for the railway reconstruction project.

According to these literature reviews, this study summarizes sources of risk in construction project as 7 risk dimensions that could affect the project success. They are financial and economic risks (*F*_1_), contractual and legal risks (*F*_2_), subcontractors-related risk (*F*_3_), operational and safety risks (*F*_4_), political and social risks (*F*_5_), design risk (*F*_6_), and force majeure risk (*F*_7_) as listed in Table 1. In addition, each of the risk dimensions is also divided into 4 to 6 factors for a railway reconstruction and the hierarchical structure of risk factors is shown in Figure 1.

### 2.2. Assessing the Railway Reconstruction Project Risk Using Fuzzy MCDM Approach

Multiple criteria decision-making (MCDM) was used to deal with the complexity and diversity of the analyses of multiple risk factors. However, it is unrealistic to assign a crisp value for a subjective judgment, especially when the information is vague or imprecise. Therefore, the analysis of this study was conducted by using the fuzzy MCDM approach that includes the implementation of fuzzy concept and MCDM approach. Fuzzy concept uses a range instead of a value to quantify the property of uncertainty and vagueness of risk factors. Furthermore, MCDM approach was used to determine the impact and possibility of risk factors. Fuzzy MCDM analysis has been widely applied to solving the problems with more than one attribute/factor having ambiguous measurement. This study measures degree of impact of risk factors and rating occurrence likelihood of occurrence of risk factors using an integrated measure of magnitude of unintentional events and impacts of events on project success [31]. Assuming the different project risk factors equally affect project success is impractical. To better manage project risks and increase chances of project success, degree of impact and occurrence likelihood of risk factors on project success should be carefully evaluated and further used as the fundamental information for the control, response, and management of project risks. That is, the varying effects of project risk factors on project success provide valuable information needed to allocate railway reconstruction project resources. Some concepts and operations of fuzzy MCDM used in this study are briefly described as the following.

#### 2.2.1. Fuzzy Number

Fuzzy numbers are a fuzzy subset of real numbers, representing the expansion of the idea of the confidence interval. Trapezoid fuzzy numbers (TFN) should possess the following basic features.

Let $\tilde{A}$ be a trapezoid fuzzy number (TFN), and its member ship function $\mu_{\tilde{A}}(x):X\to \left[0,1\right]$ is equal to

(1) $$\begin{array}{c} \mu_{\tilde{A}}\left(x\right)=\{\begin{array}{cc} 0, & x\leq a_{1}\;\;\text{or}\;x\geq a_{4},\\ \frac{\left(x-a_{1}\right)}{\left(a_{2}-a_{1}\right)}, & a_{1}\leq x\leq a_{2},\\ \frac{\left(a_{4}-x\right)}{\left(a_{4}-a_{3}\right)}, & a_{3}\leq x\leq a_{4},\\ 1, & a_{2}\leq x\leq a_{3}. \end{array} \end{array}$$

**Figure 2:**
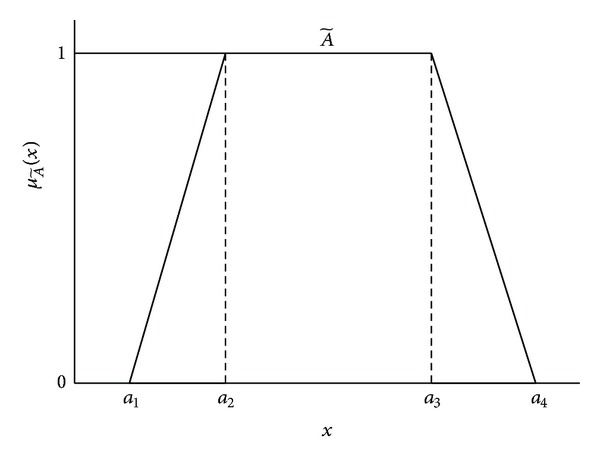
The membership function of the trapezoid fuzzy number.

The trapezoid fuzzy number can be denoted by $\tilde{A}=(a_{1},a_{2},a_{3},a_{4})$, where *a*_1_ ≤ *a*_2_ ≤ *a*_3_ ≤ *a*_4_, respectively, and *a*_1_ and *a*_4_ are the lower and upper bounds of $\tilde{A}$ (see Figure 2). The operational laws of two TFNs $\tilde{A}=(a_{1},a_{2},a_{3},a_{4})$ and $\tilde{B}=(b_{1},b_{2},b_{3},b_{4})$ are shown as below [32].

Addition of fuzzy number:

(2) A~⊕B~=(a1,a2,a3,a4)⊕(b1,b2,b3,b4)=(a1+b1,a2+b2,a3+b3,a4+b4).

Subtraction of fuzzy number:

(3) A~⊝B~=(a1,a2,a3,a4)⊝(b1,b2,b3,b4)=(a1−b4,a2−b3,a3−b2,a4−b1).

Multiplication of fuzzy number:

(4) A~⊗B~=(a1,a2,a3,a4)⊗(b1,b2,b3,b4)=(a1×b1,a2×b2,a3×b3,a4×b4).

Division of fuzzy number:

(5) A~⊘B~=(a1,a2,a3,a4)⊘(b1,b2,b3,b4)=(a1÷b4,a2÷b3,a3÷b2,a4÷b1).

Reciprocal of fuzzy number:

(6) $$\begin{array}{c} {\tilde{A}}^{-1}=\left(\frac{1}{a_{4}},\frac{1}{a_{3}},\frac{1}{a_{2}},\frac{1}{a_{1}}\right). \end{array}$$

#### 2.2.2. Linguistic Variables

**Figure 3:**
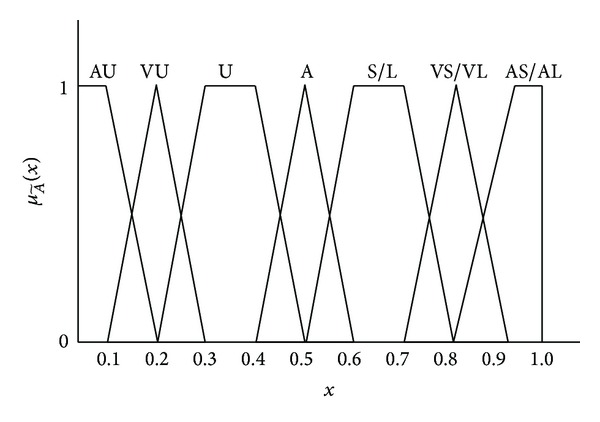
Membership functions of linguistics variables for measuring risk factors.

Zadeh [33] mentioned that it is difficult to have a logic expression in a fuzzy or vagueness environment by using a conventional quantifying approach. A linguistic variable is essentially the variable represented by a word or a sentence in human languages. This study employed the seven semantic scales to evaluate degree of impact for railway reconstruction projects and their corresponding trapezoid fuzzy numbers (TFNs) are listed in Table 2. Another seven semantic scales and their corresponding TFNs, listed in Table 3, are applied to measuring the occurrence likelihood for each risk factor in railway reconstruction projects.  Figure 3 displays the Membership functions of linguistics variables for measuring risk factors.

#### 2.2.3. Determining the Degree of Impact of Risk Factors

The matrix $\tilde{X}$ for the degree of impact of each of the risk factors (*F*_*j*_,  *j* = 1,2,…, *n*) was displayed as (7). The evaluators (*E* ^*i*^,  *i* = 1,2,…, *m*) input their subjective judgments of the degree of impact for each risk factor by using the semantic variable listed in Table 2,

(7) X~=F1F2F3⋮FnE1E2E3⋯Em[x~11x~12x~13⋯x~1mx~21x~22x~23⋯x~2mx~31x~32x~33⋯x~3m⋮⋮⋮⋱⋮x~n1x~n2x~n3⋯x~nm],i=1,2,3,…,m, j=1,2,3,…,n,

where *m* denotes the number of evaluators, *n* is the number of risk factors, and ${\tilde{x}}_{j}^{i}=(a_{1j}^{i},a_{2j}^{i},a_{3j}^{i},a_{4j}^{i})$ indicates the fuzzy degree of impact assessed by the *i*th evaluator for the *j*th risk factor.

Each of the evaluators independently performed his/her assessments based on his/her experience, intuition, and knowledge. An average score computation, displayed as (8), is then employed to synthesize the TFNs of *m* evaluators, which explored a synthesized fuzzy degree of impact value ${\tilde{w}}_{j}$ for each of the risk factors,

(8) $$\begin{array}{c} {\tilde{w}}_{j}=\frac{1}{m}\left[\sum_{i=1}^{m}{\tilde{x}}_{j}^{i}\right], \end{array}$$

where ${\tilde{w}}_{j}=(a_{1j},a_{2j},a_{3j},a_{4j})$ represents the synthesized fuzzy degree of impact of the *j*th risk factor.

The synthesized results of the fuzzy risk assessment are still in fuzzy numbers format. Therefore, it is necessary to further conduct defuzzification approach to transfer fuzzy numbers to crisp numbers. By using centroid method [34], the aggregated trapezoid fuzzy numbers (${\tilde{w}}_{j}$) were then defuzzified to the best nonfuzzy performance (BNP) values (*w*_*j*_) as the centroid value of TFNs (${\tilde{w}}_{j}$), which is displayed and proofed as (9)

(9) wj=∫a1a4x·μw~(x)dx∫a1a4μw~(x)dx=(∫a1a2x(x−a1)(a2−a1)dx+∫a2a3x dx+∫a3a4x(a4−x)(a4−a3)dx) ×(∫a1a2(x−a1)(a2−a1)dx+∫a2a31 dx+∫a3a4(a4−x)(a4−a3)dx)−1=a32+a42+a3·a4−a12−a22−a1·a23(a3+a4−a1−a2),

where *w*_*j*_ is the degree of impact of the *j*th risk factor in crisp numbers format. Finally, the normalized degree of impact of the *j*th risk factor was computed according to

(10) $$\begin{array}{c} R_{j}=\frac{w_{j}}{\sum_{j=1}^{n}w_{j}},\;\text{where}\;\sum_{j=1}^{n}R_{j}=1. \end{array}$$

#### 2.2.4. Measuring the Occurrence Likelihood of Risk Factors

The same evaluators (*E* ^*i*^, *i* = 1,2,…, *m*) as assessing degree of impact are invited to input their subjective judgments for the occurrence likelihood of each risk factor (*F*_*j*_, *j* = 1,2,…, *n*) by employing the linguistic scales listed in Table 3. The decision matrix $\tilde{Y}$ is defined as

(11) Y~=F1F2F3⋮FnE1E2E3⋯Em[y~11y~12y~13⋯y~1my~21y~22y~23⋯y~2my~31y~32y~33⋯y~3m⋮⋮⋮⋱⋮y~n1y~n2y~n3⋯y~nm],i=1,2,3,…,m, j=1,2,3,…,n,

where *m* denotes the number of evaluators, *n* is the number of risk factors, and ${\tilde{y}}_{j}^{i}=(a_{1j}^{i},a_{2j}^{i},a_{3j}^{i},a_{4j}^{i})$ indicates the occurrence likelihood of the *j*th risk factor assessed by *i*th evaluator.

Then this study uses the average score computation as (12) to synthesize the TFNs of *m* evaluators, which finally obtained a synthesized fuzzy likelihood value ${\tilde{p}}_{j}$,

(12) $$\begin{array}{c} {\tilde{p}}_{j}=\frac{1}{m}\left[\sum_{i=1}^{m}{\tilde{y}}_{j}^{i}\right], \end{array}$$

where ${\tilde{p}}_{j}=(a_{1j},a_{2j},a_{3j},a_{4j})$ represents the synthesized fuzzy likelihood regarding the *j*th risk factor. Similarly, the BNP value for the fuzzy number ${\tilde{p}}_{j}$ can also be yielded via

(13) $$\begin{array}{c} P_{j}=\frac{a_{3}^{2}+a_{4}^{2}+a_{3}\cdot a_{4}-a_{1}^{2}-a_{2}^{2}-a_{1}\cdot a_{2}}{3\left(a_{3}+a_{4}-a_{1}-a_{2}\right)}, \end{array}$$

where *P*_*j*_ is the crisp occurrence likelihood of the *j*th risk factor.

#### 2.2.5. Evaluating the Values of Degree of Risk

Once the degree of impacts and occurrence likelihood of risk factors are determined, the degree of impacts and the occurrence likelihood of each risk factor are multiplied and computed as (14) to investigate degree of risk for risk factors (*K*_*j*_):

(14) $$\begin{array}{c} K_{j}=R_{j}\times P_{j}, \end{array}$$

where *R*_*j*_ denotes the normalized degree of impact of the *j*th risk factor and *P*_*j*_ represents the occurrence likelihood of the *j*th risk factors.

#### 2.2.6. Proposed Risk Management Framework

**Figure 4:**
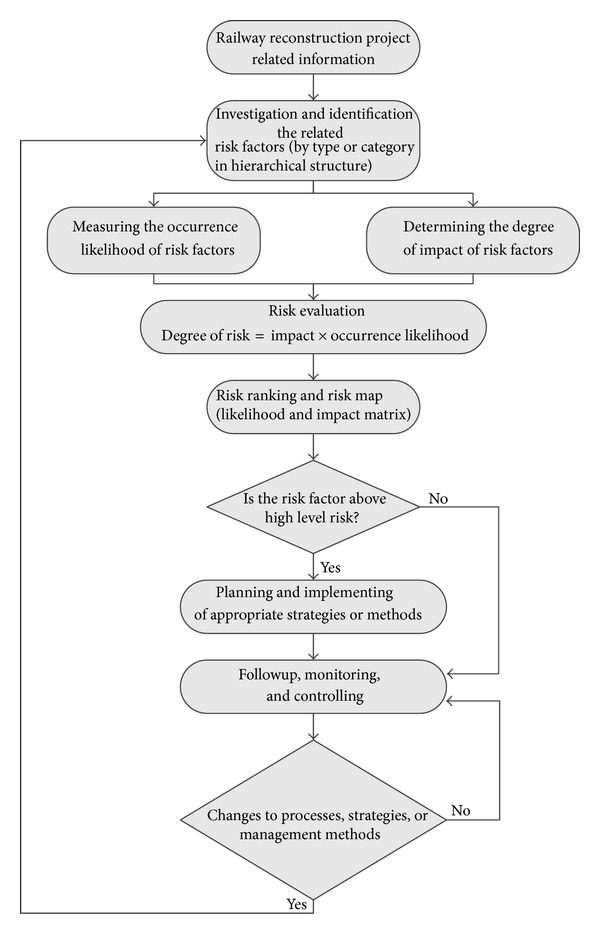
The framework of risk analysis/management for railway reconstruction project.

This study mainly applies the fuzzy multiple criteria decision-making (Fuzzy MCDM) approach to assess the hierarchical structure of risk factors for the railway reconstruction project in Taiwan. The methodology mentioned above is to establish an analytical model for measuring the degree of impact and occurrence likelihood of identified risk factors and evaluating the prediction degree of risk for each identified risk factor. The proposed risk management framework as shown in Figure 4 has the following main steps: identify risk factors, develop risk factors hierarchical structure, develop conditions of the risk factors in terms of degree of impact and occurrence likelihood, synthesize the results across hierarchy to determine relative severity of the risk factors and their risk ranking, develop a risk matrix with likelihood of risk occurrence in one axis and impact in another axis, identify risk factors and their risk level for each risky activities, and develop risk responses/strategies for each risk factor.

## 3. Empirical Case

In the empirical case of this study, 23 experts with many experiences on railway reconstruction field in Taiwan were invited to reflect their judgments on questionnaires designed to measure the degree of impact and occurrence likelihood for hierarchical structure of risk factors on railway reconstruction project. The 23 experts comprise 18 males and 5 females, in which 7 are 10–15 years experiences, 11 are 15–20 years experiences, and 5 are above 20 years experiences.

### 3.1. Degree of Impact Calculation of the Risk Factors

Seven major risk aspects, comprising 31 risk factors, are considered in this assessment case. Degree of impact for these 31 risk factors is obtained by a series of interviews with the 23 assessment representatives. The following is the computational process involved in deriving the degree of impact of risk factors using the Fuzzy MCDM approach.

1. These experts are asked to express their opinions regarding the degree of impact of each risk factor by using linguistic terms defined in Table 2. The evaluation results are listed in Tables 4 and 5.
2. In this stage, the linguistic variables are transferred into corresponding trapezoid fuzzy numbers. Since the judgments and experiences of these experts are different, (8) is used to aggregate their subjective judgments toward the degree of impact of risk factors yielding the synthesized trapezoid fuzzy numbers for each risk factor listed in Table 8.
3. Defuzzify each aggregated trapezoid fuzzy number into a crisp value for ranking and further calculation. This study adopts the defuzzification method of centroid of the normal trapezoid fuzzy number [30] to derive the BNP values of degree of impact for risk factors. The trapezoid fuzzy numbers listed in Table 8 are defuzzified by using (9), and the results are shown in the third column of Table 8.
4. Equation (10) is used to normalize the degree of impact of risk factors for each risk aspect level. Table 8 also summarizes the normalized degree of impact and ranking of each risk factor assessed by the 23 evaluators.

The results reveal that the five most significant risk factors are materials price fluctuation of financial and economic risk (*F*_13_), questionable construction site investigation of design risk (*F*_65_), funds unavailability of contractor of financial and economic risk (*F*_12_), incompatibility with original railway operations of operational and safety risk (*F*_41_), and difficulties in relocation of original pipelines of operational and safety risk (*F*_43_). Meanwhile, the five least impact risk factors include occupational safety and health regulations change of political and social risk (*F*_53_), delay payment on contract and extracontractual and legal risk (*F*_23_), wages and salaries increasing of financial and economic risk (*F*_14_), fire accident of force majeure risk (*F*_73_), and interest rate of loan increasing of financial and economic risk (*F*_11_).

### 3.2. Calculation of the Occurrence Likelihood Rating with respect to Each Risk Factor

The occurrence likelihood rating for railway reconstruction project with regard to each identified risk factor is calculated as follows.

1. To investigate and realize the actual circumstances of the project, the 23 experts are interviewed and asked to express the occurrence likelihood based on each risk factor using linguistic variables shown in Table 3, and the results of the ratings are shown in Tables 6 and 7.
2. Since the cognition of each expert varies according to their subjective intuition or experiences, after converting the linguistic variables into corresponding trapezoid fuzzy numbers, this study uses (12) to synthesize their different expressions towards the possible rating of occurrence with respect to each risk factor, deriving the aggregated trapezoid fuzzy numbers listed in Table 9.
3. Also employing the method of centroid of the normal trapezoid fuzzy number to compute the BNP value of the fuzzy possible rating with respect to each risk factor, by using (13), the trapezoid fuzzy numbers are defuzzified into crisp values and the ranking can be found in Table 9.

The results reveal that the five risk factors the most likelihood to occur are as follows: materials price fluctuation of financial and economic risk (*F*_13_), typhoon of force majeure risk (*F*_71_), difficulties in relocation of original pipelines of operational and safety risk (*F*_43_), earthquake of force majeure risk (*F*_72_), and limitations of construction sites of operational and safety risk (*F*_42_), meanwhile, the five risk factors more unlikelihood to occur are as follows: fire accident of force majeure risk (*F*_73_), delay in solving contractual issues of official matter of contractual and legal risk (*F*_21_), imperfect construction quality of operational and safety risk (*F*_45_), incomplete construction equipment of operational and safety risk (*F*_44_), and inadequate construction specifications of design risk (*F*_64_).

### 3.3. Determining the Degree of Risk

After the degree of impact of identified risk factors and the possible rating of occurrence likelihood regarding each risk factor are calculated, use (14) and multiply the degree of impact and the occurrence likelihood with respect to each identified risk factor; the estimating degree of risk and ranking for each risk factor are derived as listed in the last two columns of Table 9. As we can find the most risky factor is materials price fluctuation (*F*_13_), the second is difficulties in relocation of original pipelines (*F*_43_), the third is funds unavailability of contractor (*F*_12_), the fourth is limitations of construction sites (*F*_42_), and the fifth is typhoon (*F*_71_). These results show the properties and situations of railway reconstruction in Taiwan.

**Figure 5:**
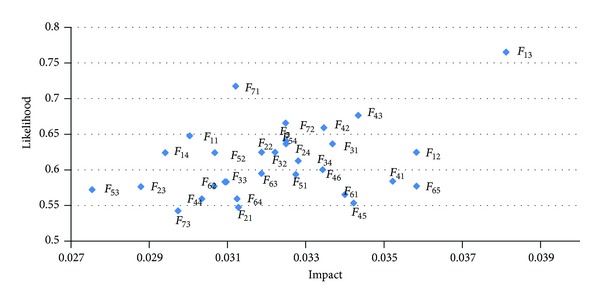
Likelihood and impact distribution diagram.

In addition, according to the BNP values of degree of impact of Table 8 and occurrence likelihood of Table 9, it can develop a likelihood and impact distribution diagram for visualizing the severity of the identified risk factors, as shown in Figure 5. The diagram also enables the related stakeholders of project to be aware which risk factors are important and their priorities.

## 4. Conclusions

Generally speaking, more preparation in advance will be less loss on operation. Thus, this study offers a simple and systemic model to evaluate project risks for a railway reconstruction project in Taiwan. Based on the results investigated following the built model to perform the risk management or plan response strategies for construction projects. This model can benefit the stakeholders of railway reconstruction project to recognize what risk factors they face and to facilitate risk assessment and furthermore complete project risk management plan. The purpose of this study is to provide a scientific and simple applied framework for risk assessment for railway reconstruction projects, and based on the built framework a decision support system for project risk management could be further developed. This study proposes a multicriteria risk factor framework through complete literature review to quantify project risks. In the built model, fuzzy concept and proposed framework were employed to quantify the qualitative attributes with subjective judgments, including the degree of impact and occurrence likelihood of risk factors. This process enables decision makers to formalize the complicated, multicriteria, and fuzzy/vague perception problem of risk assessment for railway reconstruction projects. If an integrated decision support system is developed based on the built model, it will provide more benefits for project managers in making critical decisions for railway reconstruction projects.

## Figures and Tables

**Table 1:** Related literatures for risk factors in construction project.

	*F*_1_	*F*_2_	*F*_3_	*F*_4_	*F*_5_	*F*_6_	*F*_7_
Chapman (2001) [13]	✓	✓	✓	✓	✓	✓	✓
Tah and Carr (2001) [14]	✓	✓	✓	✓	✓	✓	✓
Shen et al., (2001) [15]	✓	✓		✓	✓		
Faber and Stewart (2003) [16]				✓		✓	✓
Baloi and Price (2003) [17]	✓	✓		✓	✓	✓	✓
Ghosh and Jintanapakanont (2004) [18]	✓	✓	✓	✓	✓	✓	✓
Öztaş and Ökmen (2004) [19]	✓	✓	✓	✓	✓	✓	✓
Bing et al., (2005) [20]	✓	✓	✓	✓	✓	✓	✓
Öztaş and Ökmen (2005) [21]		✓	✓	✓		✓	
Zou et al., (2006) [22]	✓			✓		✓	✓
Lam et al., (2007) [23]	✓	✓	✓	✓	✓	✓	✓
Zou et al., (2007) [24]			✓	✓	✓	✓	
Dikmen et al., (2007) [25]	✓	✓	✓	✓		✓	
Zayed et al., (2008) [26]	✓	✓	✓	✓	✓	✓	✓
Luu et al., (2009) [27]	✓		✓	✓		✓	✓
Mojtahedi et al., (2010) [28]	✓	✓	✓	✓	✓	✓	✓
Zavadskas et al., (2010) [29]	✓		✓	✓	✓	✓	✓
Nieto-Morote and Ruz-Vila (2011) [30]			✓	✓		✓	

**Table 2:** Linguistic scales of degree of impact.

Semantic scale	Corresponding TFNs
Absolutely serious (AS)	(0.8, 0.9, 1.0, 1.0)
Very serious (VS)	(0.7, 0.8, 0.8, 0.9)
Serious (S)	(0.5, 0.6, 0.7, 0.8)
Average (A)	(0.4, 0.5, 0.5, 0.6)
Unserious (U)	(0.2, 0.3, 0.4, 0.5)
Very unserious (VU)	(0.1, 0.2, 0.2, 0.3)
Absolutely unserious (AU)	(0.0, 0.0, 0.1, 0.2)

Note: this table is revised from [32] defined.

**Table 3:** Linguistic scales of occurrence likelihood.

Semantic scale	Corresponding TFNs
Absolutely likely (AL)	(0.8, 0.9, 1.0, 1.0)
Very likely (VL)	(0.7, 0.8, 0.8, 0.9)
Likely (L)	(0.5, 0.6, 0.7, 0.8)
Average (A)	(0.4, 0.5, 0.5, 0.6)
Unlikely (U)	(0.2, 0.3, 0.4, 0.5)
Very unlikely (VU)	(0.1, 0.2, 0.2, 0.3)
Absolutely unlikely (AU)	(0.0, 0.0, 0.1, 0.2)

Note: this table is revised from [32] defined.

**Table 4:** The evaluation results of degree of impact for risk factors by *E*_1_ *∼E*_11_.

	*E*_1_	*E*_2_	*E*_3_	*E*_4_	*E*_5_	*E*_6_	*E*_7_	*E*_8_	*E*_9_	*E*_10_	*E*_11_
*F*_11_	S	S	VS	VS	A	A	A	U	S	A	U
*F*_12_	A	AS	AS	AS	VS	A	VS	A	VS	S	S
*F*_13_	VS	VS	VS	VS	S	VS	S	S	AS	S	VS
*F*_14_	VS	VS	VS	A	A	S	A	A	S	U	A
*F*_21_	A	S	S	S	S	A	A	VS	A	U	S
*F*_22_	A	A	S	VS	S	U	A	S	S	U	S
*F*_23_	A	A	S	S	A	U	A	VS	A	U	S
*F*_24_	S	A	S	AS	A	A	S	S	A	A	VS
*F*_31_	VS	U	AS	AS	VS	S	A	AS	A	S	S
*F*_32_	A	U	VS	S	S	S	A	VS	A	A	S
*F*_33_	A	A	S	VS	VS	A	U	VS	A	A	A
*F*_34_	S	A	VS	A	VS	A	A	AS	S	A	S
*F*_41_	U	VS	VS	AS	VS	S	A	AS	A	VS	VS
*F*_42_	A	VS	A	S	S	S	S	VS	VS	S	A
*F*_43_	A	VS	S	VS	S	A	A	S	VS	S	S
*F*_44_	A	S	VS	S	VS	S	A	S	A	A	S
*F*_45_	A	VS	AS	AS	VS	S	S	AS	A	A	A
*F*_46_	A	S	VS	S	VS	S	VS	AS	A	A	S
*F*_51_	S	U	A	VS	S	VS	S	AS	VS	S	U
*F*_52_	VS	A	S	S	A	A	A	S	S	A	S
*F*_53_	VS	A	S	U	A	A	A	A	S	A	U
*F*_54_	S	S	S	A	S	S	A	VS	AS	S	S
*F*_61_	A	S	AS	VS	VS	S	S	S	S	A	A
*F*_62_	A	S	VS	S	S	S	S	S	S	A	A
*F*_63_	A	VS	VS	S	S	A	A	A	A	A	A
*F*_64_	A	VS	VS	A	S	S	S	VS	A	A	A
*F*_65_	A	VS	AS	VS	S	S	A	AS	A	S	S
*F*_71_	A	S	AS	VS	S	A	S	U	A	A	A
*F*_72_	A	VS	AS	AS	S	A	VS	AS	A	S	A
*F*_73_	U	A	AS	S	VS	U	S	S	A	A	A
*F*_74_	U	U	AS	S	S	A	A	S	S	S	S

**Table 5:** The evaluation results of degree of impact for risk factors by *E*_12_~*E*_23_.

	*E*_12_	*E*_13_	*E*_14_	*E*_15_	*E*_16_	*E*_17_	*E*_18_	*E*_19_	*E*_20_	*E*_21_	*E*_22_	*E*_23_
*F*_11_	A	A	S	A	S	A	S	VS	A	A	S	S
*F*_12_	VS	S	AS	A	S	U	S	VS	AS	S	U	VS
*F*_13_	VS	VS	VS	S	VS	VS	S	VS	S	VS	S	S
*F*_14_	VS	U	A	S	A	A	A	S	A	U	A	S
*F*_21_	S	VS	S	U	S	VS	VS	A	S	A	A	S
*F*_22_	S	VS	VS	A	S	S	S	S	AS	U	A	VS
*F*_23_	S	S	A	U	A	S	VS	S	S	VU	A	S
*F*_24_	S	VS	S	U	S	S	S	S	VS	A	A	VS
*F*_31_	S	VS	VS	A	S	A	S	A	S	U	A	VS
*F*_32_	VS	VS	S	S	S	A	S	S	S	S	A	S
*F*_33_	VS	S	S	A	S	A	S	S	VS	A	U	S
*F*_34_	S	VS	VS	A	S	A	S	A	S	VS	A	S
*F*_41_	AS	AS	VS	A	A	A	S	U	VS	VS	U	VS
*F*_42_	S	S	VS	S	S	A	S	A	VS	S	S	S
*F*_43_	S	VS	S	S	S	A	S	AS	S	AS	A	S
*F*_44_	S	S	VS	U	A	A	S	U	S	A	U	S
*F*_45_	AS	AS	AS	U	A	A	VS	U	VS	S	VU	S
*F*_46_	VS	VS	VS	S	A	A	VS	A	S	A	VU	VS
*F*_51_	AS	AS	VS	U	S	S	S	VU	VS	VU	S	S
*F*_52_	AS	VS	VS	U	S	S	S	VU	S	AU	S	S
*F*_53_	S	A	S	U	A	S	A	U	S	VU	S	S
*F*_54_	VS	VS	VS	A	A	S	S	VU	VS	VU	S	S
*F*_61_	S	AS	VS	A	S	S	VS	A	AS	U	A	S
*F*_62_	S	VS	S	U	A	S	S	VU	A	S	A	S
*F*_63_	S	VS	VS	A	S	S	S	VU	S	AS	A	VS
*F*_64_	S	AS	VS	U	A	S	S	VU	VS	VU	A	VS
*F*_65_	VS	AS	AS	U	A	S	S	S	AS	S	S	VS
*F*_71_	A	AS	S	A	A	VS	A	S	S	VU	VS	S
*F*_72_	S	A	VS	U	S	VS	A	VU	VS	AU	VS	S
*F*_73_	VS	A	S	A	A	VS	A	VU	VS	AU	VS	S
*F*_74_	VS	VS	S	A	A	VS	S	U	S	U	A	S

**Table 6:** The evaluation results of occurrence likelihood for risk factors by *E*_1_~*E*_11_.

	*E*_1_	*E*_2_	*E*_3_	*E*_4_	*E*_5_	*E*_6_	*E*_7_	*E*_8_	*E*_9_	*E*_10_	*E*_11_
*F*_11_	VL	VL	L	A	L	A	L	L	L	A	L
*F*_12_	L	A	L	A	L	L	A	L	VL	L	L
*F*_13_	AL	VL	L	AL	L	VL	L	VL	AL	VL	VL
*F*_14_	AL	L	L	A	A	L	L	A	VL	L	A
*F*_21_	A	A	VL	U	A	A	A	A	L	L	L
*F*_22_	L	A	L	VL	L	A	L	VL	VL	L	L
*F*_23_	L	L	AL	VU	A	L	L	L	A	L	L
*F*_24_	L	L	VL	A	A	L	L	L	L	L	VL
*F*_31_	VL	A	L	VL	L	A	L	L	L	VL	L
*F*_32_	L	A	A	L	L	L	A	VL	A	L	VL
*F*_33_	L	U	L	A	L	A	A	VL	L	L	L
*F*_34_	L	A	A	L	L	A	A	VL	VL	L	L
*F*_41_	A	A	VL	VU	L	L	A	L	VL	VL	L
*F*_42_	L	A	L	A	VL	L	A	L	AL	VL	L
*F*_43_	A	A	L	U	VL	L	A	L	VL	VL	L
*F*_44_	L	A	A	VU	L	A	A	L	A	L	L
*F*_45_	A	A	VL	A	L	A	A	A	A	VL	L
*F*_46_	A	L	AL	A	L	L	L	L	L	L	L
*F*_51_	L	VU	A	L	VL	L	A	A	VL	AL	A
*F*_52_	VL	L	L	L	L	L	L	L	VL	VL	A
*F*_53_	L	U	L	L	L	L	L	L	VL	L	A
*F*_54_	L	L	L	L	L	VL	A	A	AL	AL	L
*F*_61_	A	A	VL	A	L	A	A	L	L	L	A
*F*_62_	L	A	VL	A	L	A	A	L	L	L	A
*F*_63_	L	A	VL	A	L	A	A	L	A	VL	A
*F*_64_	L	A	VL	U	L	A	A	L	A	L	A
*F*_65_	L	L	L	U	L	A	A	A	L	L	L
*F*_71_	L	VL	VL	AL	VL	L	L	AL	L	L	VL
*F*_72_	L	VL	VL	L	VL	L	L	AL	L	L	A
*F*_73_	A	A	VL	A	L	A	A	L	A	A	A
*F*_74_	A	A	VL	A	L	L	A	A	L	A	VL

**Table 7:** The evaluation results of occurrence likelihood for risk factors by *E*_12_~*E*_23_.

	*E*_12_	*E*_13_	*E*_14_	*E*_15_	*E*_16_	*E*_17_	*E*_18_	*E*_19_	*E*_20_	*E*_21_	*E*_22_	*E*_23_
*F*_11_	L	AL	L	A	VL	L	L	VL	L	L	L	L
*F*_12_	A	VL	L	L	VL	A	L	VL	VL	A	A	L
*F*_13_	VL	AL	VL	VL	VL	VL	L	VL	L	AL	VL	L
*F*_14_	VL	VL	L	VL	L	A	L	L	L	VU	L	L
*F*_21_	A	L	A	U	VL	L	L	A	L	VU	A	L
*F*_22_	A	L	A	A	VL	L	VL	L	VL	U	A	L
*F*_23_	A	A	U	U	VL	L	VL	L	L	U	A	L
*F*_24_	L	L	A	A	VL	L	VL	L	VL	L	A	L
*F*_31_	A	VL	VL	A	VL	L	L	A	L	L	A	L
*F*_32_	A	A	L	L	VL	L	L	L	VL	VL	A	L
*F*_33_	A	A	L	A	L	L	L	L	VL	U	A	L
*F*_34_	A	VL	VL	A	L	L	L	A	L	L	A	L
*F*_41_	AU	L	A	A	L	L	VL	U	VL	VL	A	L
*F*_42_	VL	AL	VL	VL	L	L	L	A	L	A	L	L
*F*_43_	L	AL	VL	VL	L	L	VL	AL	VL	AL	A	L
*F*_44_	A	A	VL	U	L	L	L	U	VL	L	A	L
*F*_45_	U	L	L	U	L	L	VL	U	L	U	U	L
*F*_46_	L	L	VL	A	L	L	L	A	A	VU	A	L
*F*_51_	L	AL	VL	U	VL	L	L	VU	A	U	L	L
*F*_52_	L	AL	VL	A	VL	L	L	VU	A	VU	L	L
*F*_53_	A	A	VL	A	L	L	L	U	A	AU	L	L
*F*_54_	VL	AL	L	A	L	L	L	VU	VL	U	L	L
*F*_61_	VU	L	L	L	L	L	L	A	L	U	A	L
*F*_62_	U	VL	VL	A	L	L	L	VU	L	A	A	L
*F*_63_	U	VL	VL	A	L	VL	L	VU	L	VL	A	L
*F*_64_	U	A	VL	L	L	VL	L	VU	L	U	A	L
*F*_65_	A	VL	L	A	A	A	L	L	L	A	A	L
*F*_71_	AL	AL	AL	VL	VL	VL	L	L	L	VU	L	L
*F*_72_	AL	AL	VL	VL	VL	VL	L	VU	L	AU	L	L
*F*_73_	U	L	L	L	L	VL	L	VU	A	AU	L	L
*F*_74_	A	VL	L	A	L	VL	L	U	L	U	A	L

**Table 8:** The degree of impact and its ranking for each risk factor.

	Impact TFNs	Impact BNPs	Local weights	Local ranking	Global weights	Global ranking
*F*_1_						
*F*_11_	(0.46, 0.56, 0.60, 0.70)	0.578	0.224	3	0.030	27
*F*_12_	(0.57, 0.67, 0.73, 0.81)	0.696	0.269	2	0.036	3
*F*_13_	(0.63, 0.73, 0.77, 0.87)	0.746	0.289	1	0.039	1
*F*_14_	(0.45, 0.55, 0.58, 0.68)	0.565	0.219	4	0.029	29

*F*_2_						
*F*_21_	(0.48, 0.58, 0.63, 0.73)	0.604	0.252	3	0.031	19
*F*_22_	(0.49, 0.59, 0.65, 0.74)	0.616	0.257	2	0.032	17
*F*_23_	(0.43, 0.53, 0.58, 0.68)	0.552	0.230	4	0.029	30
*F*_24_	(0.50, 0.60, 0.66, 0.75)	0.629	0.262	1	0.033	13

*F*_3_						
*F*_31_	(0.53, 0.63, 0.68, 0.77)	0.653	0.260	1	0.034	8
*F*_32_	(0.50, 0.60, 0.65, 0.75)	0.624	0.249	3	0.032	16
*F*_33_	(0.48, 0.58, 0.62, 0.72)	0.598	0.238	4	0.031	22
*F*_34_	(0.52, 0.62, 0.66, 0.75)	0.636	0.253	2	0.033	11

*F*_4_						
*F*_41_	(0.57, 0.67, 0.71, 0.79)	0.684	0.176	1	0.035	4
*F*_42_	(0.52, 0.62, 0.68, 0.78)	0.650	0.167	4	0.034	9
*F*_43_	(0.54, 0.64, 0.70, 0.79)	0.667	0.171	2	0.034	5
*F*_44_	(0.46, 0.56, 0.61, 0.71)	0.585	0.150	6	0.030	26
*F*_45_	(0.54, 0.64, 0.70, 0.77)	0.662	0.170	3	0.034	6
*F*_46_	(0.53, 0.63, 0.67, 0.76)	0.649	0.166	5	0.034	10

*F*_5_						
*F*_51_	(0.50, 0.60, 0.67, 0.76)	0.633	0.266	1	0.033	12
*F*_52_	(0.47, 0.56, 0.62, 0.72)	0.591	0.248	3	0.031	24
*F*_53_	(0.40, 0.50, 0.55, 0.65)	0.526	0.221	4	0.027	31
*F*_54_	(0.50, 0.60, 0.66, 0.75)	0.629	0.264	2	0.033	14

*F*_6_						
*F*_61_	(0.53, 0.63, 0.69, 0.78)	0.659	0.208	2	0.034	7
*F*_62_	(0.46, 0.56, 0.62, 0.72)	0.591	0.187	5	0.031	25
*F*_63_	(0.50, 0.60, 0.63, 0.73)	0.616	0.195	3	0.032	18
*F*_64_	(0.49, 0.59, 0.62, 0.72)	0.603	0.191	4	0.031	20
*F*_65_	(0.57, 0.67, 0.73, 0.81)	0.696	0.220	1	0.036	2

*F*_7_						
*F*_71_	(0.48, 0.58, 0.63, 0.72)	0.602	0.251	2	0.031	21
*F*_72_	(0.51, 0.61, 0.65, 0.74)	0.628	0.262	1	0.032	15
*F*_73_	(0.46, 0.55, 0.59, 0.69)	0.572	0.238	4	0.030	28
*F*_74_	(0.47, 0.57, 0.63, 0.73)	0.597	0.249	3	0.031	23

**Table 9:** The occurrence likelihood and degree of risk for each risk factor.

	Likelihood TFNs	Likelihood BNPs	Likelihood ranking	Degree of risk	Risk ranking
*F*_1_					
*F*_11_	(0.53, 0.63, 0.70, 0.79)	0.662	6	0.020	17
*F*_12_	(0.51, 0.61, 0.66, 0.76)	0.637	10	0.023	3
*F*_13_	(0.67, 0.77, 0.82, 0.90)	0.787	1	0.031	1
*F*_14_	(0.51, 0.61, 0.67, 0.76)	0.636	13	0.018	22

*F*_2_					
*F*_21_	(0.43, 0.53, 0.57, 0.67)	0.552	30	0.017	27
*F*_22_	(0.51, 0.61, 0.66, 0.76)	0.637	11	0.020	14
*F*_23_	(0.45, 0.55, 0.62, 0.71)	0.583	24	0.017	29
*F*_24_	(0.52, 0.62, 0.68, 0.78)	0.650	8	0.021	9

*F*_3_					
*F*_31_	(0.53, 0.63, 0.67, 0.77)	0.650	9	0.022	6
*F*_32_	(0.51, 0.61, 0.66, 0.76)	0.637	12	0.020	15
*F*_33_	(0.46, 0.56, 0.62, 0.72)	0.591	20	0.018	23
*F*_34_	(0.50, 0.60, 0.65, 0.75)	0.624	15	0.021	13

*F*_4_					
*F*_41_	(0.47, 0.57, 0.61, 0.71)	0.593	19	0.021	11
*F*_42_	(0.55, 0.65, 0.70, 0.80)	0.673	5	0.023	4
*F*_43_	(0.57, 0.67, 0.72, 0.81)	0.692	3	0.024	2
*F*_44_	(0.44, 0.54, 0.59, 0.69)	0.565	28	0.017	28
*F*_45_	(0.43, 0.53, 0.59, 0.69)	0.559	29	0.019	21
*F*_46_	(0.48, 0.58, 0.64, 0.74)	0.610	16	0.021	12

*F*_5_					
*F*_51_	(0.48, 0.58, 0.63, 0.72)	0.602	18	0.020	16
*F*_52_	(0.51, 0.61, 0.67, 0.76)	0.636	14	0.020	18
*F*_53_	(0.45, 0.54, 0.61, 0.71)	0.580	25	0.016	31
*F*_54_	(0.52, 0.62, 0.69, 0.78)	0.653	7	0.022	8

*F*_6_					
*F*_61_	(0.44, 0.54, 0.60, 0.70)	0.572	26	0.019	19
*F*_62_	(0.46, 0.56, 0.61, 0.71)	0.585	23	0.018	25
*F*_63_	(0.49, 0.59, 0.62, 0.72)	0.604	17	0.019	20
*F*_64_	(0.44, 0.54, 0.59, 0.69)	0.565	27	0.018	26
*F*_65_	(0.46, 0.56, 0.61, 0.71)	0.585	22	0.021	10

*F*_7_					
*F*_71_	(0.61, 0.71, 0.77, 0.85)	0.735	2	0.023	5
*F*_72_	(0.56, 0.65, 0.71, 0.80)	0.680	4	0.022	7
*F*_73_	(0.43, 0.52, 0.57, 0.67)	0.547	31	0.016	30
*F*_74_	(0.47, 0.57, 0.61, 0.71)	0.591	21	0.018	24
