# Corrigendum to “Using Fuzzy Multiple Criteria Decision Making Approach for Assessing the Risk of Railway Reconstruction Project in Taiwan”

**DOI:** 10.1155/2020/4712104

**Published:** 2020-06-12

**Authors:** Shih-Tong Lu, Shih-Heng Yu, Dong-Shang Chang

**Affiliations:** ^1^Graduate Institute of Project Management, Kainan University, No. 1 Kainan Road, Luchu, Taoyuan 33857, Taiwan; ^2^Department of Business Administration, National Central University, No. 300 Jhongda Road, Jhongli, Taoyuan 32001, Taiwan

In the article titled “Using Fuzzy Multiple Criteria Decision Making Approach for Assessing the Risk of Railway Reconstruction Project in Taiwan” [1], the authors did not cite their closely related conference article published in 2010 [2] and would like to cite two earlier sources by other authors [3, 4] that used similar wording in the methods.

The conference article [2] presented a concept and framework for this research, which was proposed using the triangular fuzzy number, whereas the trapezoid fuzzy number was used in [1] for more accuracy in linguistic variables, and full and complete expert questionnaires were also used. The theoretical descriptions and computational process statements of fuzzy multiple criteria decision making (Fuzzy MCDM) theory and the general description of the mathematical formulae and background literature are the same, as well as the conclusions, but the description of the research is more complete.

Chang and Wang [3] and Chen and Hwang [4] should have been cited in the theoretical descriptions and computational process statements of Fuzzy MCDM theory and the general description of the mathematical formulae in Sections 2.2, 2.2.1, and 3.1, and [3] should have been cited in Section 3.2.

The editorial board agreed to the publication of a corrigendum.
